# Enhancing hydrodynamic forces through miniaturized control of square cylinders using the lattice Boltzmann method

**DOI:** 10.1038/s41598-024-65423-4

**Published:** 2024-07-05

**Authors:** Ahmed Refaie Ali, Waqas Sarwar Abbasi, Rabia Younus, Hamid Rahman, Sumaira Nadeem, Afraz Hussain Majeed, Irshad Ahmad

**Affiliations:** 1 Department of Mathematics and Computer Science, Faculty of Science, Menoufia University, Shebin El Kom 32511, Menofia, Egypt; 2 Department of Mathematics, Air University, Islamabad, 44000 Pakistan; 3 Department of Mathematics and Statistics, Women University Swabi, Swabi, 23430 Pakistan; 4 Department of Medical Rehabilitation Sciences, College of Applied Medical Sciences, King Khalid University, Abha, Saudi Arabia; 5 School of Energy and Power Engineering, Jiangsu University, Zhenjiang 212013, China

**Keywords:** Lift, Suppression, Vorticity. Miniaturized control cylinders, Hydrodynamic forces, Regimes, Lattice Boltzmann Method (LBM), Drag coefficient, Mechanical engineering, Applied mathematics, Computational science, Applied physics, Fluid dynamics

## Abstract

This study investigates the influence of small control cylinders on the fluid dynamics around a square cylinder using the Lattice Boltzmann Method (LBM). Varying the gaps (L) between the main and control cylinders from 0 to 6, four distinct flow regimes are identified: the solo body regime (SBR), shear layer reattachment (SLR), suppressed fully developed flow (SFDF), and intermittent shedding (IS). The presence of control cylinders results in significant reductions in flow-induced forces, with drag coefficient (CD) and root mean square values of drag and lift coefficients (*CD*_*rms*_ and *CL*_*rms*_) decreasing by approximately 31%, 90%, and 81%, respectively. The SFDF flow regime exhibits the lowest fluid forces compared to other regimes. The effects of tiny control cylinders on the fluid flow characteristics of a square cylinder are examined using the Lattice Boltzmann Method (LBM) in this research work. The gaps (L) between the main and control cylinders are varied in the range from 0 to 6. The size of each control cylinder is equal to one-fifth of the primary cylinder. According to the findings, there are four distinct flow regimes as the gap spacing varies: solo body regime (SBR), shear layer reattachment (SLR), suppressed fully developed flow (SFDF), and intermittent shedding (IS) for gap spacing ranges 0 ≤ L ≤ 0.2, 0.3 ≤ L ≤ 0.9, 1 ≤ L ≤ 3, and 3.2 ≤ L ≤ 6, respectively. Additionally, it has been noted that the amplitude of variable lift force is reduced when the gap separation between the main and control cylinders is increased. When compared to solo cylinder values, it is found that the presence of small control cylinders in the flow field results in a considerable reduction of flow-induced forces. The SFDF flow regime was determined to have the lowest fluid forces compared to the other flow regimes studied. Our findings highlight the efficacy of small control cylinders in mitigating flow-induced forces and controlling flow characteristics. The LBM proves to be a valuable computational technique for such fluid flow problems.

## Introduction

The interaction between fluid and solid objects is of great concern for many engineering problems. The direct sources of concern in this interaction are fluctuating forces and vortex shedding phenomena, which may result in vibration and oscillation of structures. Vortices are shed from the sides of structures as the wind blows over them. In such situations, low-pressure zones are formed on the structure’s rear side, causing forces (drag and lift) to fluctuate and act at different angles corresponding to the wind direction. Vortex shedding may be responsible for the generation of resonance phenomena in high-rise buildings, piers of bridges in channel beds, and other relevant problems. This makes it imperative to keep an eye out for flow-induced impacts to considerably enhance engineering design.

There have been several studies in the past many decades addressing such problems. These studies consider the flows around cylinders (circular/rectangular) as prototypes of flows around civil structures. Park et al.^1^ analyzed the flow past a circular block at $Re$ = 160 by considering different resulting flow quantities including $St$, $CD,$ and $CL$, base pressure, separation angle, and length of the separation bubble. Norberg^2^ studied the aspects of fluctuating lift resulting from the flow across a circular block for $Re$ wide range from 47 to 2.2 × 10^5^. He concluded that the initial shedding of the vortices occurred at $Re$ = 47 and the $C{L}_{rms}$ increased rapidly within the laminar shedding regime. Davis and Moore^3^ considered numerical simulations of 2D steady flow about rectangles in infinite domains for $Re$ = 100–2800 in their study. They reported strong dependence of *Cd*, *Cl,* and *St* on $Re$ with variations in rectangle dimension, upstream velocity profile, and angle of attack. Ahmad et al.^4^ calculated $St$ of rectangular cylinders for $Re$ ranging from 75 to 150. They found sharp changes in $St$ for various aspect ratios of the cylinder at $Re$ = 145 and 150. Perumal et al.^5^ computationally studied the fluid flow across a square obstacle because of $40\le Re\le 150$. They found that the channel length, blockage ratio, and Reynolds number have a considerable consequence on the fluid flow features. Islam et al.^6^ studied fluid flow past a square cylinder at $Re$ = 150 for several up and down-stream locations with blockages. They found that the parameters such as $CD$, $C{D}_{rms}$, $C{L}_{rms}$, $C{D}_{m}$ and $St$ considerably affected by these domain locations. Patil and Tiwari^7^ investigated the vortex shedding phenomenon for flow across a transversally vibrating square block. They compared the natural vortex shedding frequency of a fixed square cylinder with the situation where a square cylinder has an excitation frequency. The critical $Re$ and Strouhal frequency (${f}_{s}$) were determined by Thompson et al.^8^ for elliptical cylinders of different aspect ratios ($A$) (from circular to flat plate case). In the case of flat plates, wake vortices were found to be stronger and larger. In the case of a circular cylinder, a decrease in $A$ caused an increase in the circulations of the wake. Some further studies addressing such problems can be found in^9,10^ and references there within.

From the literature, one can also find several studies regarding the control of wake and suppression of flow-induced forces using different control strategies (active and passive control strategies). Some of those are being discussed here. The effect of two detachable regulating rods with varied gaps ( ${g}_{1}$, ${g}_{2}$) on the flow around a square cylinder at a constant Re = 160 was calculated by Manzoor et al.^11^. They found three different types of flow modes under the effect of ${g}_{1}$ and ${g}_{2}$. To establish the optimal plate configuration, The fluid forces acting on a cylinder were investigated by Abdi et al.^12^ using a setup with several rigid splitting plates mounted to the rear of the cylinder. It was discovered that increasing the number of splitter plates might reduce the amount of drag force, in addition to the frequency of vortex shedding and the variable lift force. The impacts of a detached splitter plate on the vortex-shedding mechanism from a square cylinder were investigated by Kumar et al.^13^ at $Re$= 150 and 200. They concluded that by positioning the splitter plate at the back of the cylinder, the values of $CD$ and $St$ decreased while the value of $CL$ increased. Barman and Bhattacharyya^14^ reported the reduction of both $CD$ and ${f}_{s}$ due to dual splitter plates for flow past a square cylinder. Abograis and Alshayji^15^ analyzed the reduction of fluid forces around a square cylinder using control plates at different positions in the flow field. They discovered that the CD and CL on the primary cylinder could be drastically reduced by using both upstream and downstream adjustment plates. In the case of the downstream control plate, vortex shedding was controlled behind the cylinder which resulted in lowering $St$. Wang et al.^16^ investigated the effect of dual-attached splitter plates on the wake transition of the flow around a circular cylinder. They considered two different attachment angles ($2{0}^{o}$ and $4{0}^{o}$) and found that due to the presence of the dual splitter plates the onset of secondary instabilities was significantly controlled. Mansy et al.^17^ investigated the effects of placing a splitter plate upstream of a square cylinder by considering different velocity ratios. An increase in $CD$ and ${f}_{s}$ was observed by increasing the velocity ratio while the splitter plate showed stabilizing effects at low-velocity ratios. Ghadimi et al.^18^ investigated the optimal control of vortex shedding from a square cylinder by a detached splitter plate. They found that at a distance L = 1.5 between cylinder and plate, the vortex shedding was suppressed. Assi et al.^19^ conducted research on the process of controlling flow around a circular cylinder that was encircled by eight revolving wake control cylinders. According to their findings, control cylinders with smaller diameters were able to suppress the main cylinder’s vortex-shedding mechanism.

Additionally, by speeding up the rotation of the control cylinders, $CD$,$CL,$ and $C{L}_{rms}$ were decreased, and the downstream separated flow region of the main cylinder was reduced. A similar attempt was also performed by Islam et al.^20^ on flow control around a square cylinder using multiple control cylinders placed at different positions with gaps from 0.5 to 8. They observed a maximum of 50.8% reduction in the average drag coefficient and 86.4% reduction in the rms value of the lift coefficient.

The study of fluid dynamics, particularly near incompressible laminar flows, has evolved significantly through various computational methods. Guo et al.^21^ compared the Lattice Boltzmann Equation (LBE) method and the Gas-Kinetic Scheme (GKS), highlighting their strengths and applications. Foundational work by Bhatnagar, Gross, and Krook^22^ introduced the BGK model for collision processes in gases, laying the groundwork for modern computational fluid dynamics (CFD) methods. Viggen’s^23^ thesis extended the Lattice Boltzmann Method (LBM) to acoustics, demonstrating its versatility. Mohammad^24^ provided a comprehensive guide on LBM fundamentals and engineering applications. Studies by Islam et al.^25^ and Abbasi et al.^26–28^ explored force statistics, wake structures, and flow control around square cylinders, enhancing the understanding of flow behavior and optimization strategies. Ali et al.^29^ and Vinodh and Supradeepan^30^ investigated flow modification techniques and control cylinder influences, offering practical insights for engineering applications.

Further integrating fluid dynamics with other fields, Abo-Seida et al.^31^ examined the Cherenkov FEL reaction in a plasma-filled waveguide, bridging fluid dynamics and plasma physics. El-Dabe et al.^32^ and Mahmuda Maya et al.^33^ explored magnetohydrodynamic (MHD) flows and convection in various contexts, highlighting the complex interactions between thermal, magnetic, and fluid effects. These studies collectively advance the understanding of fluid dynamics, providing valuable insights for applications ranging from traditional fluid mechanics to advanced thermal management and electromagnetic wave propagation systems. Gul et al.^34^ conducted a numerical investigation to explore the flow features of two horizontal rectangular polygons, providing insights into hydrodynamic behaviors. In a subsequent study, Gul et al.^35^ conducted an extensive examination of flow characteristics for two vertical rectangular polygons subjected to two-dimensional cross flow conditions, contributing further to the understanding of fluid dynamics around geometric structures.

While previous studies have investigated the flow characteristics around control cylinders in various flow regimes, including high Reynolds number flows, there remains a lack of comprehensive understanding of their behavior under low Reynolds number conditions. The majority of existing literature focuses on high Reynolds number flows, neglecting the unique flow phenomena and challenges associated with low Reynolds number regimes. Therefore, our study aims to bridge this gap by providing novel insights into the hydrodynamic interactions and flow characteristics of control cylinders at Reynolds number 100, a regime that has received limited attention in the literature.

Our research addresses the need for a systematic investigation of the hydrodynamic behavior of control cylinders in low Reynolds number flows and aims to fill the existing research gap by conducting a detailed numerical analysis. We justify the significance of our study by highlighting the importance of understanding flow behavior at low Reynolds numbers, particularly in engineering applications such as microfluidics, biofluidics, and low-speed aerodynamics, where such flows are prevalent.

To achieve our research objectives, we employ advanced computational fluid dynamics (CFD) simulations to model the flow around control cylinders and analyze the resulting hydrodynamic forces and flow characteristics. By systematically varying relevant parameters and conducting a parametric study, we aim to elucidate the influence of key factors on the hydrodynamic performance of control cylinders and provide insights into optimal design strategies for enhancing their effectiveness in controlling fluid flows.

Although various studies concerning fluid flow analysis and flow control around cylinders can be found in the literature, many aspects of this field of study have not yet been thoroughly explored. One of these features is controlling flow across a square block using small control cylinders, which is the main aim of the current study. The purpose of this study is to extend the understanding of suppression of flow-induced forces and vortex shedding control across a square cylinder using small control cylinders. In particular, this study will focus on the effects of small control cylinders placed at different positions on the fluid flow characteristics of a square cylinder. The flow structure mechanism for this geometry will be compared with that of a square cylinder without control cylinders to observe and discuss the differences in detail. Moreover, the influence of the gaps between the control cylinders and the main cylinder on the vortex-shedding mechanism and fluid forces variation will be analyzed.

The novelty of our research lies in several key aspects that collectively contribute to advancing the current understanding of low Reynolds number flow behavior and its implications for engineering applications. These include:

1. Exploration of Low Reynolds Number Regime: While previous studies have extensively investigated the hydrodynamic behavior of control cylinders in high Reynolds number flows, our research focuses specifically on the low Reynolds number regime (Re = 100). This represents a novel and underexplored area of study, considering the unique flow phenomena and challenges associated with low Reynolds number flows.
2. Comprehensive Numerical Analysis: We employ advanced computational fluid dynamics (CFD) simulations to conduct a systematic investigation of the hydrodynamic interactions and flow characteristics of control cylinders at Reynolds number 100. By utilizing state-of-the-art numerical techniques and parametric studies, we are able to provide detailed insights into the flow behavior around control cylinders in low Reynolds number flows.
3. Identification of Unique Flow Phenomena: Our study identifies and analyzes the emergence of distinct flow phenomena that are characteristic of low Reynolds number flows. These include laminar flow patterns, vortex shedding behavior, and the influence of viscous effects on flow dynamics. By elucidating these phenomena, we contribute to a deeper understanding of the fundamental principles governing fluid flow at low Reynolds numbers.
4. Practical Implications for Engineering Applications: The insights gained from our research have direct relevance to various engineering applications requiring precise flow control, such as microfluidics, biofluidics, and low-speed aerodynamics. By providing guidelines for the design and optimization of control strategies based on our numerical findings, we offer practical implications for improving the performance and efficiency of devices operating in low Reynolds number environments.

In summary, our paper represents a novel contribution to the field by addressing important research gaps, providing original insights into low Reynolds number flow behavior, and offering practical implications for engineering applications. By combining advanced numerical simulations with a focus on the low Reynolds number regime, we aim to advance the state-of-the-art in flow control and enhance our understanding of fluid dynamics in engineering systems.

### Computational scheme

In the present work, we utilized the Lattice Boltzmann Method (LBM), a widely recognized computational technique for solving fluid flow problems. LBM originated from Lattice Gas Automata (LGA) and subsequently from the direct discretization of the renowned Boltzmann equation (BE) ^21^. The Boltzmann equation without external force is:

1 $$\frac{\partial f}{\partial t}+c.\nabla f=\Omega$$

where *f* represents the distribution function that denotes the positions of particles at a particular time *t*, *c* indicates the velocity direction, and ΩΩ is the collision operator, a complex quantity due to its integro-differential form. This term cannot be simply overlooked, as the effect of a collision between two bodies is unlikely to have a major impact on the values of several measurable quantities. However, it is possible to make an approximation without significantly affecting the result. Bhatnagar, Gross, and Krook (BGK) ^22^ developed a simplified collision operator, given as:

2 $$\Omega =\omega ({f}^{eq}-f)=\frac{1}{\tau }({f}^{eq}-f)$$

where ${f}^{eq}$ is the equilibrium distributive function and $\tau$ > ½ is the relaxation time parameter.

The discretized form of Eq. (1) with substitution of Eq. (2) is:

3 $$f_{i} \left( {x + c_{i} \Delta t,t + \Delta t} \right) = f_{i} \left( {x,t} \right) + \frac{\Delta t}{\tau }\left[ {f_{i}^{eq} \left( {x,t} \right) - f_{i} \left( {x,t} \right)} \right]; i = 0,1,2, \ldots ,8$$

Note that Eq. (3) serves as an alternative to the well-known Navier–Stokes equations for solving fluid flow problems ^23^. The role of the equilibrium distribution function is crucial as it governs the behavior of the model. It takes different forms for different problems, and for the two-dimensional nine-velocity particles model (D2Q9), it has the following form:

4 $${f}_{i}^{eq}=\rho {w}_{i}[1+3({c}_{i}.{\varvec{u}})+\frac{9}{2}({c}_{i}.{\varvec{u}}{)}^{2}-\frac{3}{2}{\varvec{u}}.{\varvec{u}}]$$

where $\rho$ is the density, ${w}_{i}$ are particular weights for each particle (see Table. 1) and* u* is the macroscopic velocity. Density and momentum, among other macroscopic quantities, may be calculated using the following formulas:

**Table 1:** Weighting factors for D2Q9 lattice.

Direction $i$	Weighting factor ${w}_{i}$
$0$	$4/9$
$\text{1,2},\text{3,4}$	$1/9$
$\text{5,6},\text{7,8}$	$1/36$

5 $$\rho \left(x, t\right)=\sum_{i}{f}_{i}\left({\varvec{x}},t\right)$$

6 $$\rho \left(x, t\right){\varvec{u}}(x,t)=\sum_{i}{{{\varvec{e}}}_{i}f}_{i}\left(x,t\right)$$

**Figure 1:**
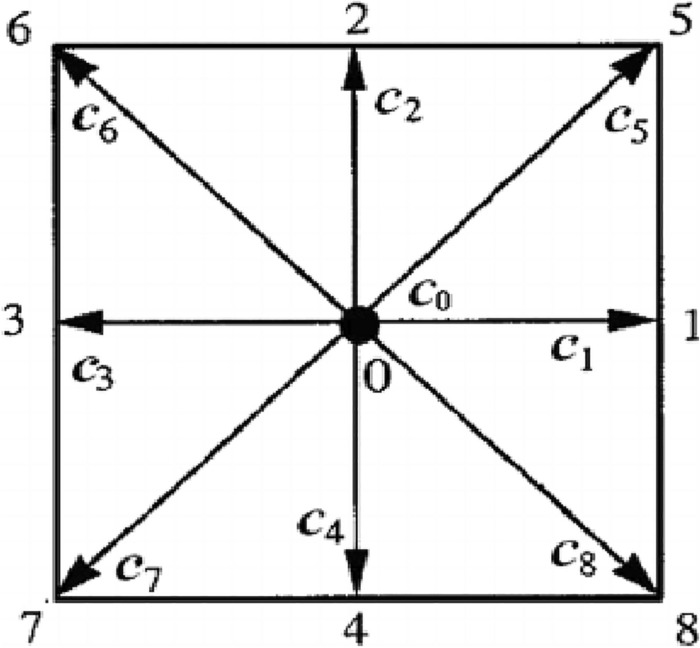
D2Q9 lattice model.

In LBM different models are used to solve problems at different dimensions ^24^. For this work, we have employed the D2Q9 model which is a very famous model for two-dimensional problems, see Fig. 1. The velocity ${c}_{0}$ corresponds to the rest particles while ${c}_{1}...{c}_{8}$ are the velocities with directions pointing towards eight neighboring nodes.

## Problem description

**Figure 2:**
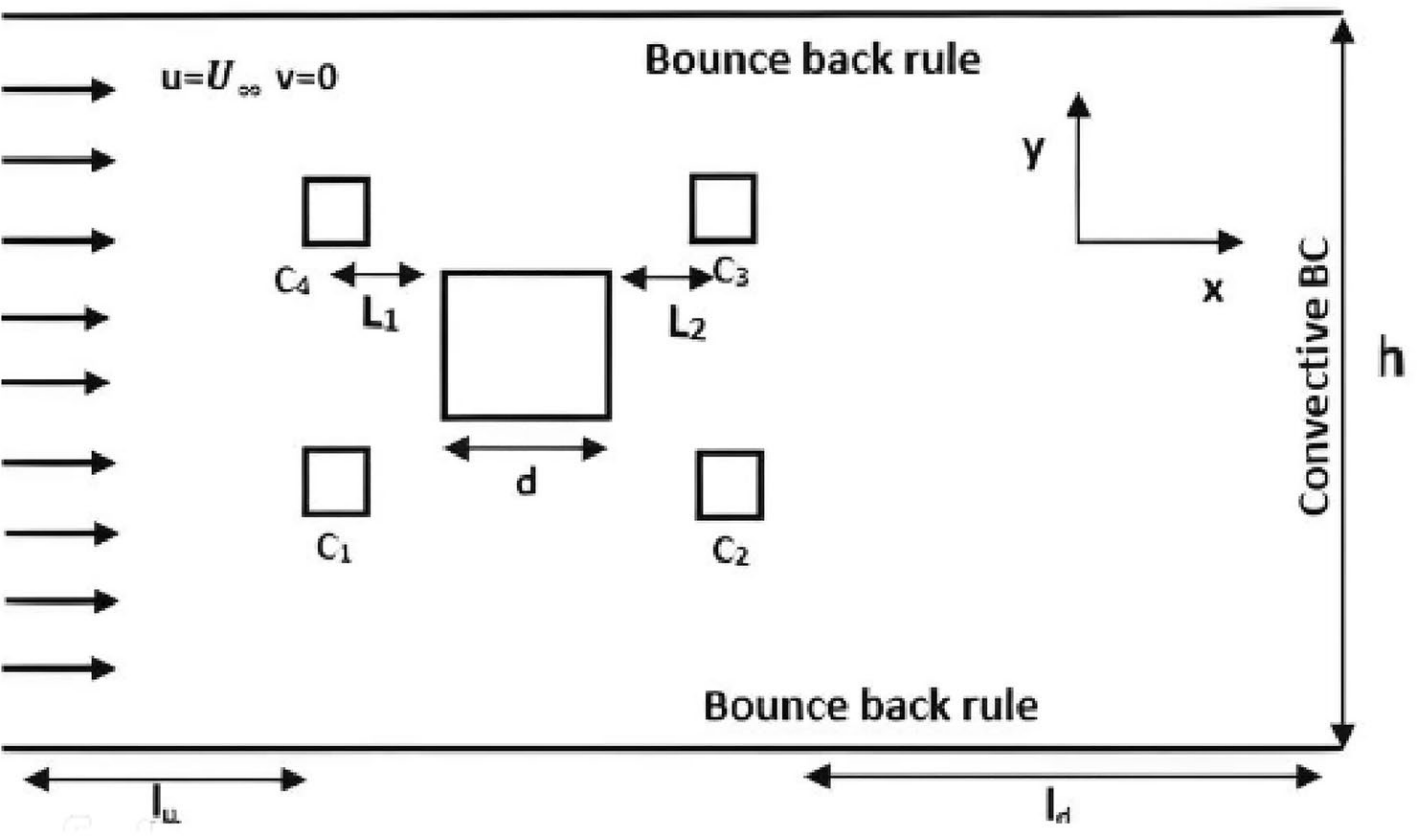
Flow chart of the problem.

The computational domain consists of a square obstacle, representing the main cylinder, with a side length of d = 20, positioned within a 2D channel. The channel has an upstream length of ${l}_{u}$ = 7 $d$ and a downstream length of ${l}_{d}$ = 20 $d$. Additionally, the height of the channel is $h$ = 21 $d$, as illustrated in Fig. 2. Four small control cylinders, each with a diameter of 0.2d, are strategically placed around the main cylinder. The distances between the main cylinder and the control cylinders, denoted as L, vary from 0 to 6d. Specifically, ${L}_{1}$ represents the distance between control cylinders ${C}_{1}$ and ${C}_{4}$ from the main cylinder, ${L}_{2}$ represents the distance between control cylinders ${C}_{2}$ and ${C}_{3}$ from the main cylinder. The gap between ${C}_{1}$,${C}_{4}$, and ${C}_{2}$,${C}_{3}$ is fixed at 1*d*. The computational domain boundaries, including the channel walls and cylinder surfaces, are set to no-slip boundary conditions using a bounce-back rule ^24^. A uniform inflow velocity is applied at the inlet, while a convective boundary condition ^6^ is imposed at the outlet of the channel. All lengths in the computational domain are dimensionless, using the size of the main cylinder (d) as the reference length.

### Grid independence study and code validation

**Figure 3:**
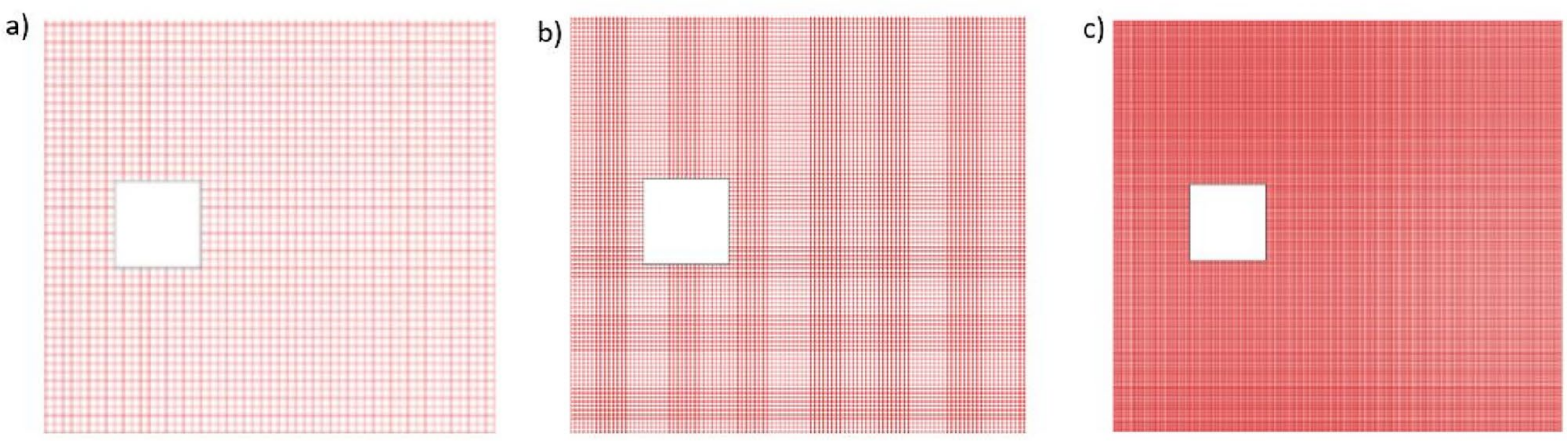
Different grid sizes for flow past a square cylinder (**a**) 10-points, (**b**) 20-points, and (**c**) 40-points.

In numerical computations, a grid independence analysis determines the optimal grid size. The selection of the grid is crucial for obtaining accurate results, as computational outcomes heavily rely on it. In this study, we employed three distinct grid sizes (with 10, 20, and 40 grid points) along the surface of the cylinder, as illustrated in Fig. 3.

The purpose behind the utilization of $C{D}_{rms}$ values in our study stems from the need to comprehensively assess the flow characteristics and aerodynamic performance of the system under investigation. $C{D}_{rms}$, or the root mean square (RMS) value of the drag coefficient fluctuations, serves as a valuable metric for quantifying the level of flow unsteadiness or turbulence-induced fluctuations in the drag force experienced by the cylinder.

In our research, we utilized three different grid sizes (10-, 20-, and 40-grid points) along the surface of the cylinder to assess grid independence. This approach allowed us to evaluate the influence of grid resolution on key physical parameters such as drag coefficient (CD), Strouhal number (St), and root mean square values of drag and lift coefficients (*CD*_*rms*_ and $C{L}_{rms}$). Our analysis revealed significant differences in the computed parameters across the different grid sizes. Specifically, we observed that the values of CD, St, $C{D}_{m}$, and $C{L}_{rms}$ varied noticeably between the 10-, 20-, and 40-point grid sizes. Notably, the values obtained at the 20-point grid size were sufficiently close to those at the 40-point grid size, indicating convergence. However, it is important to note that while the 40-point grid size provided slightly better results, it also incurred higher computational costs due to longer simulation times.

Moreover, our choice of a 20-point grid size aligns with recommendations from previous studies by Guo et al. and Manzoor et al., who suggested a grid size of 20 points for convergence when dealing with similar flow configurations around square cylinders. This consensus further supports the appropriateness of our grid size selection.

To validate our computational approach, we also conducted a code validation exercise by analyzing the flow characteristics around a single square cylinder. This step ensured that our in-house developed code accurately captured the fluid dynamics of the problem under consideration.

Therefore, we have considered the grid size investigation in our study and selected a grid size of 20 points based on convergence criteria and previous research recommendations. This choice strikes a balance between computational efficiency and accuracy, enabling us to obtain reliable results for our analysis of flow regimes and the effects of control cylinders on hydrodynamic forces.

Analysis of physical parameters is carried out for various selected grid point values at $Re$ = 100 to select an accurate grid size for better accuracy of results. Different physical parameters such as $C{D}_{m}$ ,$St$ and $C{D}_{rms}$ are computed corresponding to these different grid sizes (see Table 2). The values of these constraints are much different at a 10-point grid from those at 20- and 40-point grid size and these values lack accuracy as it is a well-known fact that the addition of more grid points improves the accuracy. Furthermore, from Table 2 it is evident that the values at 20-points grid size are sufficiently closer to those at 40-points grid size. Although 40-points grid size gives better results as compared to 20- and 10-points it is computationally expensive since the simulation takes a long time to converge. Similarly, Guo et al.^21^ and Manzoor et al.^11^ suggested a grid size of 20 points for convergence when dealing with a square cylinder.

**Table 2:** In Re = 100, comparing physical attributes across grid locations.

Parameters	10-points	20-points	40-points
$C{D}_{m}$	1.325 (6.7%)	1.400 (1.4%)	1.4201
$St$	0.144 (0.1%)	0.145 (0.4%)	0.1446
$C{L}_{rms}$	0.271 (17.3%)	0.230 (0.4%)	0.2311

### Code validation

When analyzing the flow parameters around multiple bodies, it is common practice to first investigate the flow over a single obstacle. To validate our in-house developed code, we have also computed the flow characteristics around a single square cylinder. For this purpose, we analyzed the vorticity contour, streamlines behavior, time distinctions of CD, CL, and power spectrum of the lift coefficient at Re = 100.

**Figure 4:**
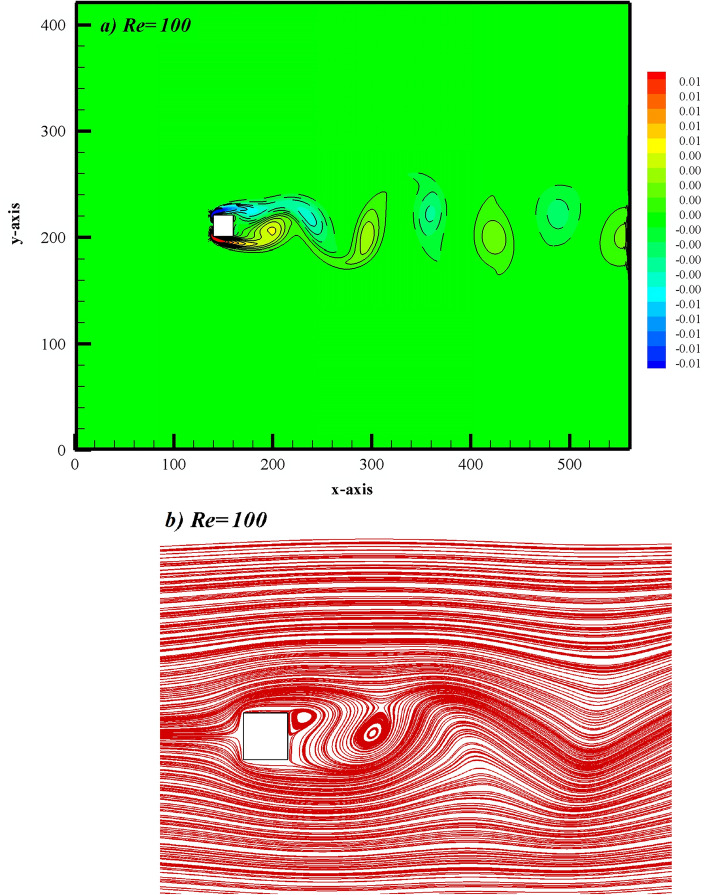
(**a**) Vorticity (**b**) Streamlines graphs for flow problem.

Figure 4a depicts the vorticity graphs, where solid lines represent positive vortices (anticlockwise rotation) generated from the lower corner of the cylinder, while dashed lines represent negative vortices (clockwise rotation) generated from the upper corner. This figure illustrates how the shear layers detached from both corners of the cylinder merge to generate vortices in the near wake. These vortices, with varying widths and sizes, alternately move in the downstream wake region, exhibiting behavior similar to a von Karman vortex street. It is a well-known fact that at Re = 100, the flow around the square cylinder becomes unsteady, with the complete development of vortices, as observed in our case. These results are consistent with those reported in the works of [3, 4, and 6].

Figure 4b shows the streamlines patterns at a specific instant in the computational domain. The periodicity of the flow observed in Fig. 4a is clearly demonstrated by the variation in streamlines. The waviness in streamlines in the far wake region and the presence of recirculation zones in the near wake provide evidence of unsteady flow. A similar pattern of streamlines can be observed in the work of Perumal et al.^5^.

**Figure 5:**
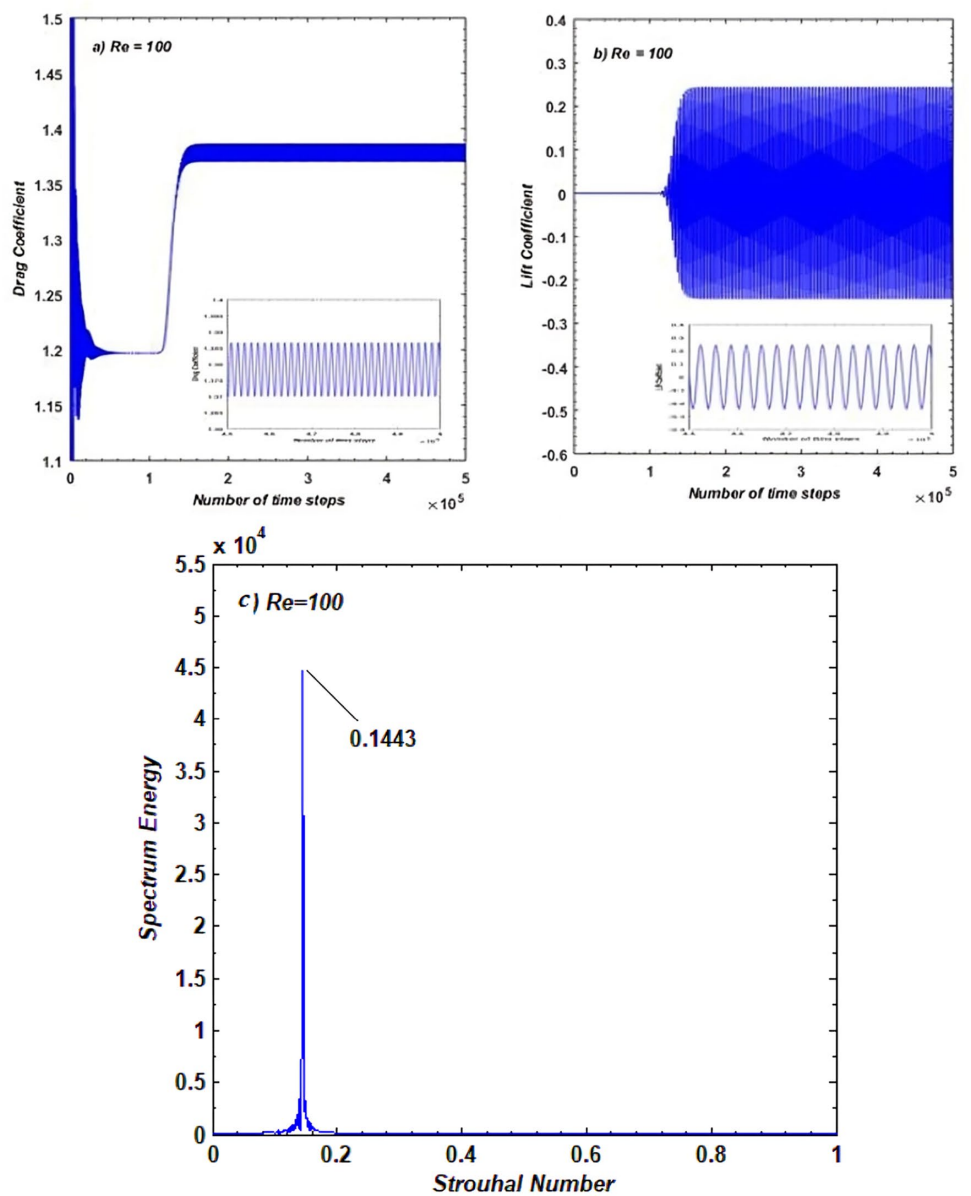
(**a**,**b**) Variation on *CD* and *CL* against time (**c**) Power spectrum for flow past a square cylinder.

The resulting variation of $CD$ and $CL$ with time, corresponding to unsteady flow observed in Fig. 4, is exposed in Fig. 5(a, b). In the case of $CD$, a jump can be seen from lower to higher values indicating irregular variations initially. The steady periodic behavior (see zoomed view) starts after approximately 1.5 × 10^5^-time steps in $CD$. The periodic nature of the lift coefficient can be seen after approximately 1 × 10^5^ time steps demonstrating the unsteady nature of the flow. The amplitude of $CL$ indicates the higher magnitude of force at $Re$ = 100 resulting from alternate shed vortices in the wake of a cylinder. Islam et al.^25^ also reported such type of variations of $CD$ and $CL$ in their numerical experiments. The power spectrum of $CL$ is offered in Fig. 5c against the *St*. The single peak in the spectrum is representing the dominance of vortex shedding frequency. Because of the uniform formation of vortices no secondary cylinder interaction frequencies were recorded from the graphs. Such spectrum is mostly observed at $Re$ = 100^3–5^.

These results indicate a good qualitative agreement between our findings and those of other researchers. For further details regarding the validation of our code, readers are referred to our published work^26–28^.

## Results and discussions

This section presents the analysis of the effects of four detached control cylinders, placed at different positions, on the fluid flow features of the main square block corresponding to different gap spacings. These effects are addressed in terms of vorticity, streamline patterns, velocity profiles, and drag coefficients. The goal is to understand how the placement and size of the control cylinders influence the flow behavior around the main square block and to identify optimal configurations for flow control. $CD,$ and $CL$, as well as the energy spectra of $CL$. Additionally, the influence that gap has on the fluctuation of some parameters, such as the average $CD$, $St$, and $rms$ values of $CD$ and $CL$, is explored. The basic purpose of placing small control cylinders is to suppress the vortex-shedding mechanism and to reduce the fluctuating forces exerted by a fluid on the square cylinder.

1. **Variables Considered and Omitted**: In our parametric studies, we considered various geometric and flow parameters, including the diameter and spacing of control cylinders, as well as the flow velocity and Reynolds number. These variables were chosen based on their significance in influencing the hydrodynamic behavior of the system and their relevance to practical engineering applications. However, it is important to acknowledge that certain factors, such as surface roughness effects, thermal gradients, and three-dimensional flow phenomena, were omitted due to their relatively minor impact in the low Reynolds number regime and the focus of our study on two-dimensional simulations. We have updated the manuscript to clearly state the variables considered and omitted, providing readers with a comprehensive understanding of the analysis and its limitations.
2. **Handling of Turbulence and Selection of Low Reynolds Numbers**: In our simulations, turbulence was not explicitly modeled, as the flow conditions considered corresponded to low Reynolds numbers (Re = 100), where laminar flow assumptions are generally valid. The decision to focus on low Reynolds numbers was motivated by the desire to investigate the hydrodynamic behavior of control cylinders under conditions relevant to microfluidic and low-speed aerodynamic applications, where laminar flow prevails. While higher Reynolds number regimes could introduce turbulence effects and alter the flow dynamics, our study aimed to establish a fundamental understanding of flow control mechanisms in the laminar flow regime. Future work could explore the extension of our analysis to higher Reynolds numbers, incorporating turbulence modeling techniques to assess its impact on flow behavior and control effectiveness.

Therefore, we have addressed your inquiries regarding the variables considered, limitations of two-dimensional analysis, handling of turbulence, and selection of low Reynolds numbers in our manuscript. We believe that these clarifications enhance the comprehensibility and rigor of our research findings, providing valuable insights for future studies in fluid dynamics and engineering applications.

Thank you for your valuable feedback, and we have incorporated these discussions into the revised version of the manuscript.

The novelty of our work lies in the investigation of utilizing miniaturized control cylinders to enhance hydrodynamic forces in the flow around a square cylinder. While previous studies have explored the influence of various geometric configurations and flow conditions on fluid forces, our research uniquely focuses on the introduction of small control cylinders to manipulate flow characteristics. Specifically, we identify four distinct flow regimes based on the gap spacing between the main and control cylinders, shedding light on the intricate interplay between these elements and the resulting hydrodynamic forces. Additionally, we employ the Lattice Boltzmann Method (LBM), a computational technique known for its efficiency and accuracy in simulating fluid flow, to conduct our analysis. By quantifying the effects of control cylinders on drag coefficient, Strouhal number, and root mean square values of drag and lift coefficients, we demonstrate the significant reduction in fluid forces achieved through this innovative approach. Overall, our work contributes to advancing the understanding of flow control mechanisms and offers valuable insights for optimizing the design of structures subjected to fluid flow.

### Flow regimes

**Figure 6:**
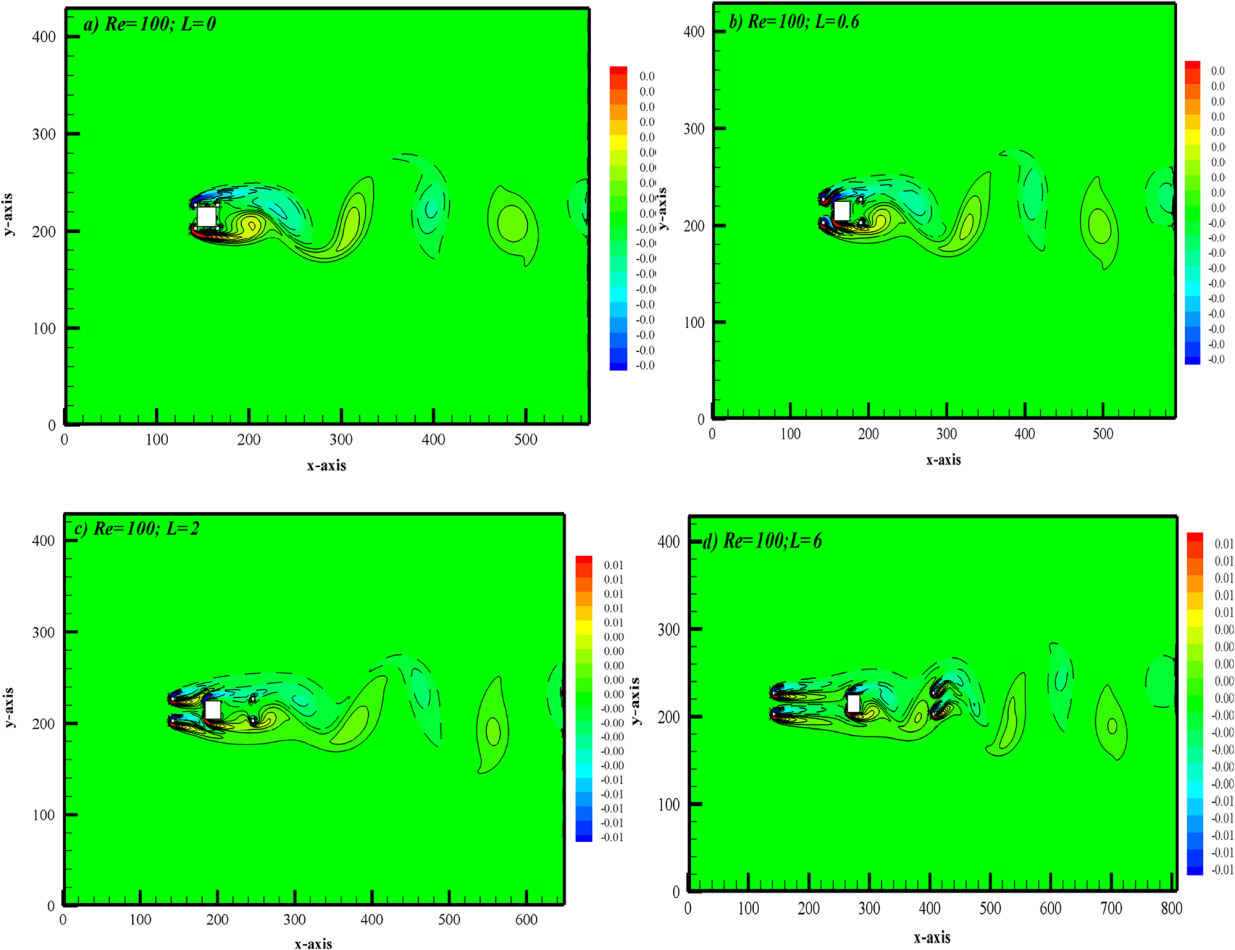
Representative vorticity contours corresponding to different flow regimes.

The present work examines various flow regimes by placing control cylinders at different locations. These flow regimes are categorized based on specific characteristics, which are discussed below:

At smaller gap spacings ranging from $L$ = 0 to 0.2, the flow structure resembles that of a solo body. A representative vorticity contour for this flow regime is shown in Fig. 6a at $L$ = 0. These images demonstrate the separation of shear layers from the controlling cylinders, placed upstream, overshooting and engulfing the main cylinder as well as the small control cylinders placed downstream. It is possible to observe alternating vortex shedding at the downward wake of the control cylinders located on the rear portion of the main cylinder as a result of the rolling up of the shear layers at the closest downstream location. Because of the close proximity of the two cylinders, there were no vortices that formed in the gap between the main cylinder and the control cylinders. All bodies behave like solo bodies because vortices shed at the down wake region only. Due to this resemblance the flow regime is named the SBR. Another flow regime analyzed is named the shear layer reattachment (SLR) for spacing range $L$ = 0.3 to 0.9. A typical instance of this flow regime is revealed in Fig. 6b at $L$ = 0.6. During this flow regime, the shear layers that split from the small control cylinders upstream rejoin at the front surface of the primary cylinder and move toward the corners. Upon detaching from the corners of the main cylinder, inner shear layers move within the gaps and reattach to the downstream control cylinders. This occurs because the fluid finds space and moves inside the cylinder gaps but is still unable to roll up into vortices. In the SLR regime, the wake formation region shortens, and the shape and size of vortices change compared to the SBR regime. As the gap ratio further increases, the flow regime transitions to the suppressed fully developed flow (SFDF), as observed in the vorticity contour in Fig. 6c at L = 2 (a representative case). It's noteworthy that this flow regime occurs over the spacing range L = 1 to 3. In this regime, the width of the wake region is suppressed, while the length increases compared to SBR and SLR.

The shear layers that move inside the gap between the main and downstream control cylinders exhibit unsteadiness, yet no vortex formation can be observed within the gaps. It appears that the upstream control cylinders act as a shield for the main cylinder, exerting some control over the fluid flow. Assi et al.^19^ noted that the placement of small diameter control cylinders around the main cylinder assists in controlling the vortex shedding mechanism. Another flow regime occurs for the spacing range L = 3.2 to 6, termed intermittent shedding (IS), as described by its representative case in Fig. 6d at L = 6. In this regime, the flow appears steady in the first gap region due to the presence of control cylinders, while vortices shed in the second gap due to sufficient space. Moreover, there is no definite size and shape of vortices, unlike in the SBR, SLR, and SFDF flow regimes. The weakening effect of control cylinders, placed in the wake, on the flow characteristics of the main cylinder is evident due to the ample gap space. This irregularity in vorticity behavior is one reason why the flow regime is termed intermittent shedding. Manzoor et al.^11^ categorized flow structures as shear layer reattachment, steady flow mode, and semi-developed vortex shedding in their study of flow around a square cylinder with dual control plates. Similarly, Ali et al.^29^ demonstrated significant alterations in the flow configuration around a square cylinder, particularly in the near wake, when a splitter plate is used. They divided the flow around a square cylinder into three distinct regimes based on the length of the connected splitter plate. Similarly, in this study, the presence of control cylinders alters the fluid flow characteristics around the main cylinder, akin to the effects observed with a splitter plate.

**Figure 7:**
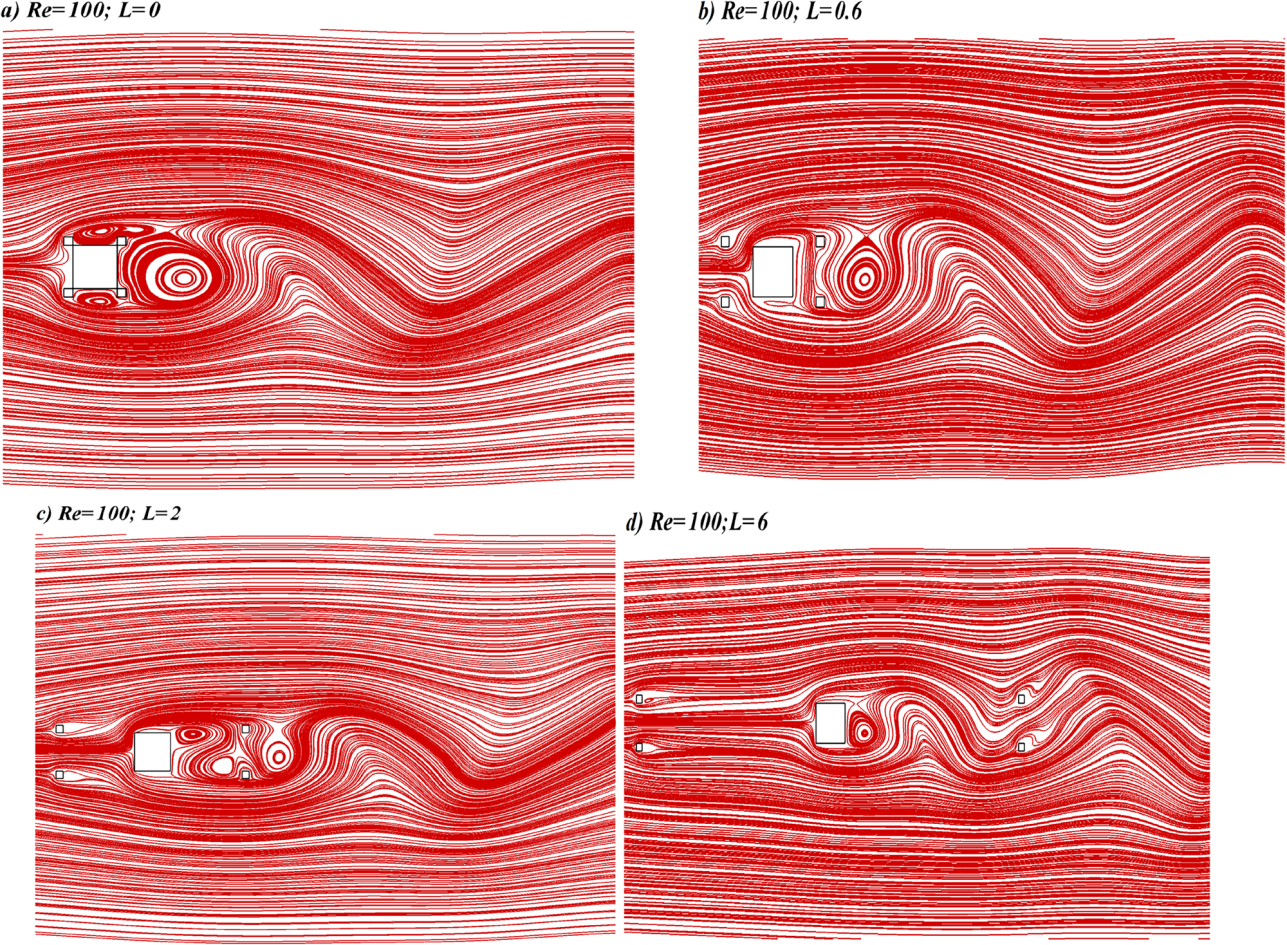
Representative instantaneous streamline patterns corresponding to different flow regimes.

Streamlines visualization corresponding to SBR flow at $L$ = 0 is shown in Fig. 7a. This figure shows that the position of the stagnation point, where the pressure remains maximum and which normally lies at the center of the front side of the main cylinder without additional bodies, is altered due to the presence of control cylinders. Instead of hitting the main cylinder the fluid first interacts with control cylinders present at the upstream location. Three eddies are shaped at the rear, upper, and lower sides of the main cylinder. In the wake of the cylinder, the wave-like behavior of streamlines result from the swirling motion of the vortices. The streamlines visualization corresponding to the SLR regime is presented in Fig. 7b. In this case, the eddies formed at the upper and lower sides of cylinders disappear which indicates some stabilizing effect of upstream control cylinders. The boundary layer formed at the front side of the main cylinder separates at corners and the shear layers reattach to downstream placed control cylinders. Streamlines visualization for the SFDF regime shows that the eddies also appear between the main cylinder and control cylinders placed downstream indicating the unsteady flow in this gap unlike SBR and SLR regimes (see Fig. 7c). This is due to an increase in gap spacing. Corresponding to IS regime the waviness of streamlines in between the primary and controlling cylinders placed downstream indicates the vortex generation in this region (Fig. 7d). At the first gap, streamlines indicate a smooth flow behavior while at the second gap oscillations in flow can be observed.

**Figure 8:**
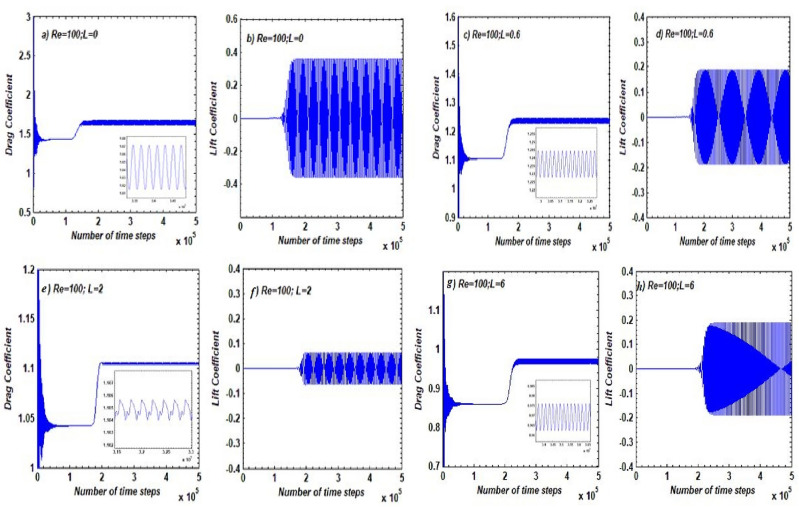
Impacts of L on *CD* and *CL*.

The corresponding CD and CL behavior against time for different flow regime cases discussed earlier is shown in Fig. 8. At L = 0, both CD and CL appear to be periodic due to vortex formation seen in SBR flow (see Fig. 8a, b and Fig. 6a). Initially, CD exhibits random oscillations followed by a constant line for some time, which can be attributed to initial variations in flow. After approximately 1.5 × 10^5^ time steps, steady periodic behavior can be seen in CD, while CL starts variations after approximately 1 × 105 time steps. Note that in the case of the solo cylinder, CD and CL showed almost similar behavior, indicating that the control cylinders have not altered the behavior of the forces at L = 0 (see Fig. 5a, b). In the case of the SLR flow regime, both CD and CL show periodicity due to unsteady flow in the downstream region but at a relatively delayed time compared to the solo body case (see Fig. 8b). It is evident from the amplitudes of CD and CL that as the distance between the main cylinder and control cylinders increases, the magnitude of both force coefficients decreases slightly, indicating that the control cylinders are gradually controlling the flow-induced forces. Figure 8c manifests that the control cylinders are much more effective at gap spacing range L = 1 to 3, where the SFDF flow regime was observed. Both CD and CL start periodicity after noteworthy time steps compared to SBR and SLR flow regimes. Furthermore, smaller amplitudes of oscillations indicate a reduction in drag force as well as lift force. The suppression in wake width and reduction in fluid forces are the main reasons why this regime is classified as SFDF. In contrast to the SFDF regime, the magnitude of CD and CL again rises as the distance between the main and control cylinders further increases. This can be observed from the time-dependent variations of CD and CL corresponding to the IS regime presented in Fig. 8d at L = 6. With increasing L, the wake interference effects weaken; thus, after suppression, the amplitudes of oscillations of CD and CL again increase due to irregularity in flow. According to Vinodh and Supradeepan^30^, the effect of the control cylinder on fluid forces almost vanishes beyond L = 3. Note that the initial steady behavior of CD is prolonged to more time steps compared to SFDF flow, but the amplitude of CL has increased in the case of the IS regime. Due to such irregularities in flow structure and force variation, this flow regime is named IS. It is worth mentioning that the zoomed view of CD is also presented in Fig. 8a, c, e, g for a clear understanding of its behavior. According to Abograis and Alshayji^15^, a combination of controlling devices placed at both upstream and downstream locations of the main cylinder is more helpful in reducing CD and CL.

**Figure 9:**
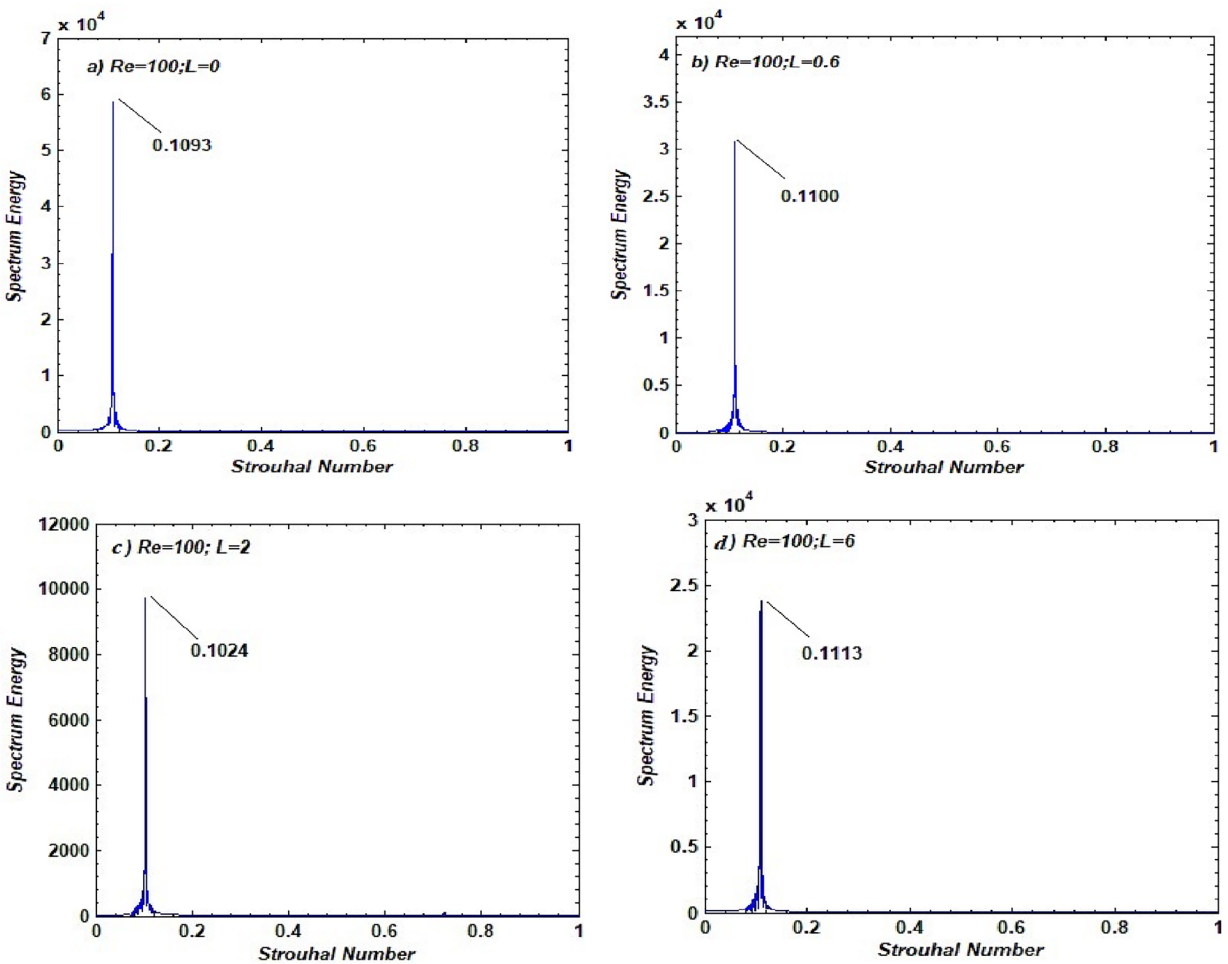
Energy spectrum of lift coefficients corresponding to different flow regimes.

The spectrum energy graphs of the CL for different lengths of plates, and hence the flow regimes, are given in Fig. 9 along with the corresponding Strouhal numbers (peak value). The sharp single peak in all graphs indicates the unsteady periodic nature of CL without any distortion due to the occurrence of fully developed vortex shedding. The magnitude of the spectrum is higher at L = 0, ensuring that the vortices behind the square cylinder are fully developed and strong in the SBR (see Fig. 9a). The energy spectrum of the lift force at L = 0.3, corresponding to the SLR regime, is shown in Fig. 9b. Due to the stabilizing effect of control cylinders, the magnitude of spectrum energy falls slightly. This can be attributed to a decrease in the amplitude of CL as the gap spacing increases. Conversely, the Strouhal number increases as the length between the main and the control cylinder increases. The spectrum energy of the lift force at L = 2, corresponding to the SFDF regime, is presented in Fig. 9c. As gap spacing increases, the reduction in Strouhal number as well as the magnitude of spectrum energy can be clearly seen, which is due to a decrease in the amplitude of CL. In contrast, the spectrum energy of the lift force at L = 6, corresponding to the IS regime, increases again, indicating the inadequacy of control cylinders at larger spacing values. Additionally, the increase in Strouhal values is due to a rise in CL amplitude resulting from the irregularity of flow (see Fig. 9d).

### Analysis of variation in physical parameters

**Figure 10:**
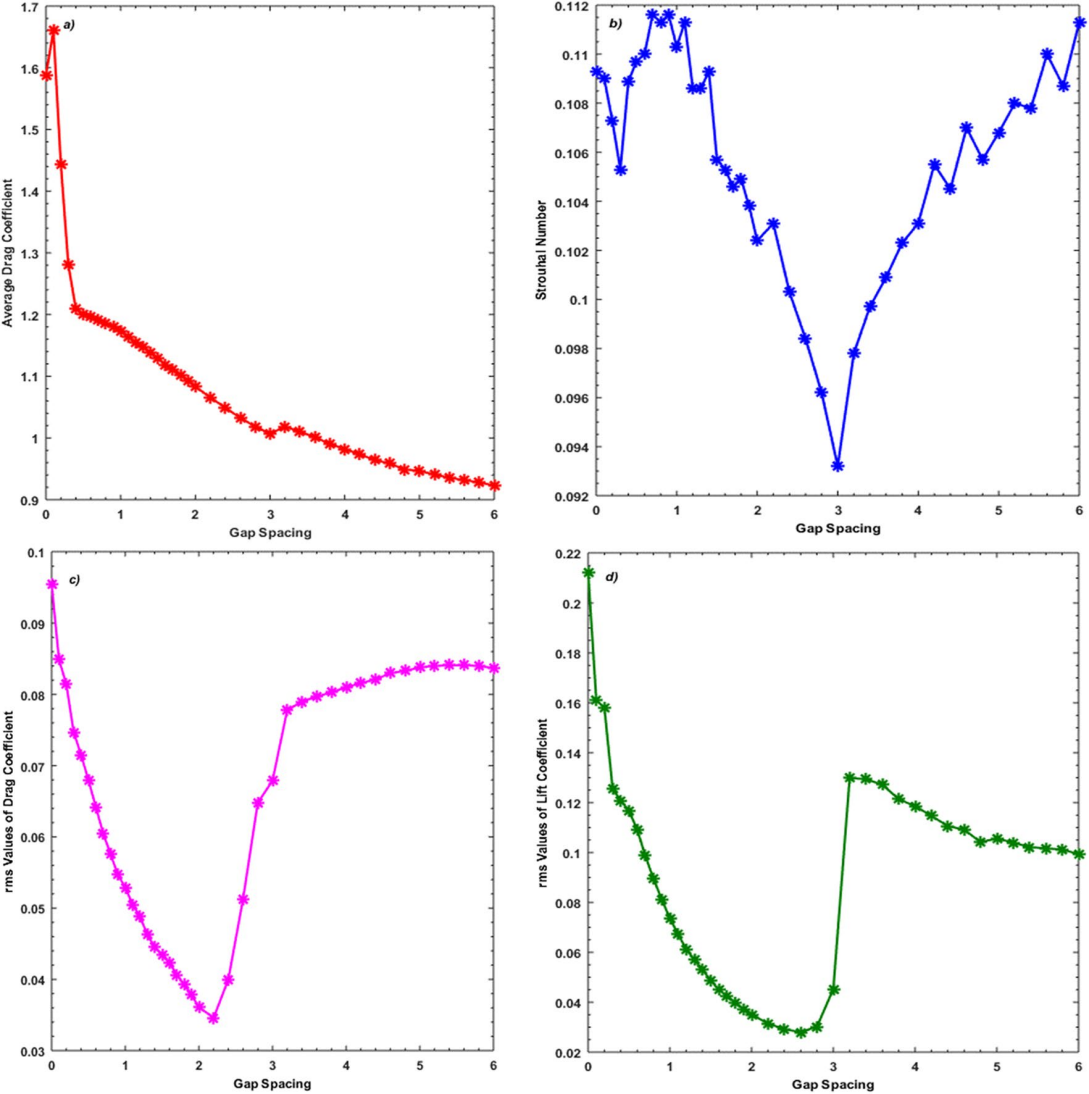
Variation on (**a**) *CDm* (b) *St* (**c**) *CDrms* (**d**) *CLrms* against gap spacing.

The variation of physical parameters like $C{D}_{m}$, $St$, $C{D}_{rms}$ and $C{L}_{rms}$ under the influence of gap spacing between the primary and controlling cylinders is presented in Fig. 10a–d. It can be examined that the highest value obtained for $C{D}_{m}$ is 1.6600 at $L$ = 0.1 (Fig. 10a) and this gap, the SBR flow was witnessed. Because of the small gap spacings, cylinders act like an extended single body which is why the drag force is higher at smaller spacing values. As the gap value increases from 0.3 to 3, the flow switched to the SLR regime, where a decrease in the values of $C{D}_{m}$ can be noticed. This decrease in $C{D}_{m}$ continues till gap spacing value $L$ = 6. Note that in the range of gap spacing 1 to 3 the SFDF regime was observed where the rapid decrease in average drag force can be observed. This is because, in this spacing range, maximum suppression of wake width was observed. At $L$ = 3.2 the $C{D}_{m}$ value slightly increases due to the switching of the flow regime from SFDF to IS regime. The variation in $C{D}_{m}$ indicates that the control cylinders significantly affect the drag force exerted by the fluid on the main cylinder. The least value 0.9231 of $C{D}_{m}$ can be seen at $L$ = 6 which indicates that the drag force much deteriorates at this spacing value. Islam et al. ^20^ also observed a reduction in $C{D}_{m}$ at high gap spacing where the vortices were entirely controlled resulting in fluid forces reduction. The variation in $St$ against gap spacings is presented in Fig. 10b. The $St$ values decrease initially for the gap spacing range $L$ = 0 to 0.3 where mainly the SBR flow was observed. After that, the $St$ values increase in the SBR range (0.3 < $L$ $\le$ 0.9). In this flow regime, the shear layers detached from upstream bodies and reattached to the downstream ones which results in a change in lift force and influencing $St$. In this range, the maximum value of $St$ = 0.1116 can be seen at $L$ = 0.9. A mixed trend of growing and decreasing behavior can be seen between gaps 1 to 2.4 where the SFDF regime was observed. In general, the rapid drop in $St$ can be observed in the SFDF flow regime ranging over 1 $\le$ $L$ $\le$ 3. This is because, in this flow regime, the amplitude of $CL$ cycles was dropped which results in the reduction of $St$. The minimum value of $St$ can be seen at $L$ = 3 which is 0.0932. After $L$ = 3, the $St$ values rise again and a mixed trend of increase and minor decrease can be observed for $L$ = 3 to 6 where IS regime was seen. This can be attributed to the chaotic nature of flow where irregularities do occur. Overall the local maximum value of $St$ is observed in SLR flow at $L$ = 0.9 and the local minimum value is 0.0932 occurs at $L$ = 3 in the SFDF regime. Islam et al. ^20^, obtained the largest value of $St$ at $L$ = 6 due to high fluid forces and a high shedding frequency, while the lowest value of $St$ was obtained in their work at $L$ = 3 due to weakly induced vortex shedding. Figure 10c shows variation in $C{D}_{rms}$ against gap spacings between the main and control cylinders. The local maximum value of 0.0955 of $C{D}_{rms}$ can be seen at $L$ = 0 where SBR flow was observed. The $C{D}_{rms}$ decrease strictly till it reaches the local minimum value of 0.0400 as gap spacing rises from 0 to 2.2. Note that at this spacing value, the SFDF regime was observed. In the range from $L$ = 2.4 till 6, the increase in $C{D}_{rms}$ values can be observed where SFDF and IS flow regimes were seen. An almost similar trend can be observed for $C{L}_{rms}$, as was seen for $C{D}_{rms}$, against gap spacings till $L$ = 2.6 (Fig. 10d). At $L$ = 0, the local maximum value of $C{L}_{rms}$ = 0.2124 is attained. After $L$ = 3, $C{L}_{rms}$ jumps to a relatively higher value but in the range of 3.2 to 6, it decreases contrary to $C{D}_{rms}$ where IS flow regime was observed.

### Analysis of physical parameter reduction

To check the effect of control cylinders on parameters like $C{D}_{m}$, $C{D}_{rms}$, $C{L}_{rms}$ and $St$ the percentage difference values of these parameters are computed from the single cylinder without control cylinders (SCWC). The relation used for the percentage difference is:

$$\%\text{Difference}= \frac{\text{SC without control cylinders }-\text{SC with control cylinders}}{\text{SC without control cylinders}}\times 100$$

**Figure 11:**
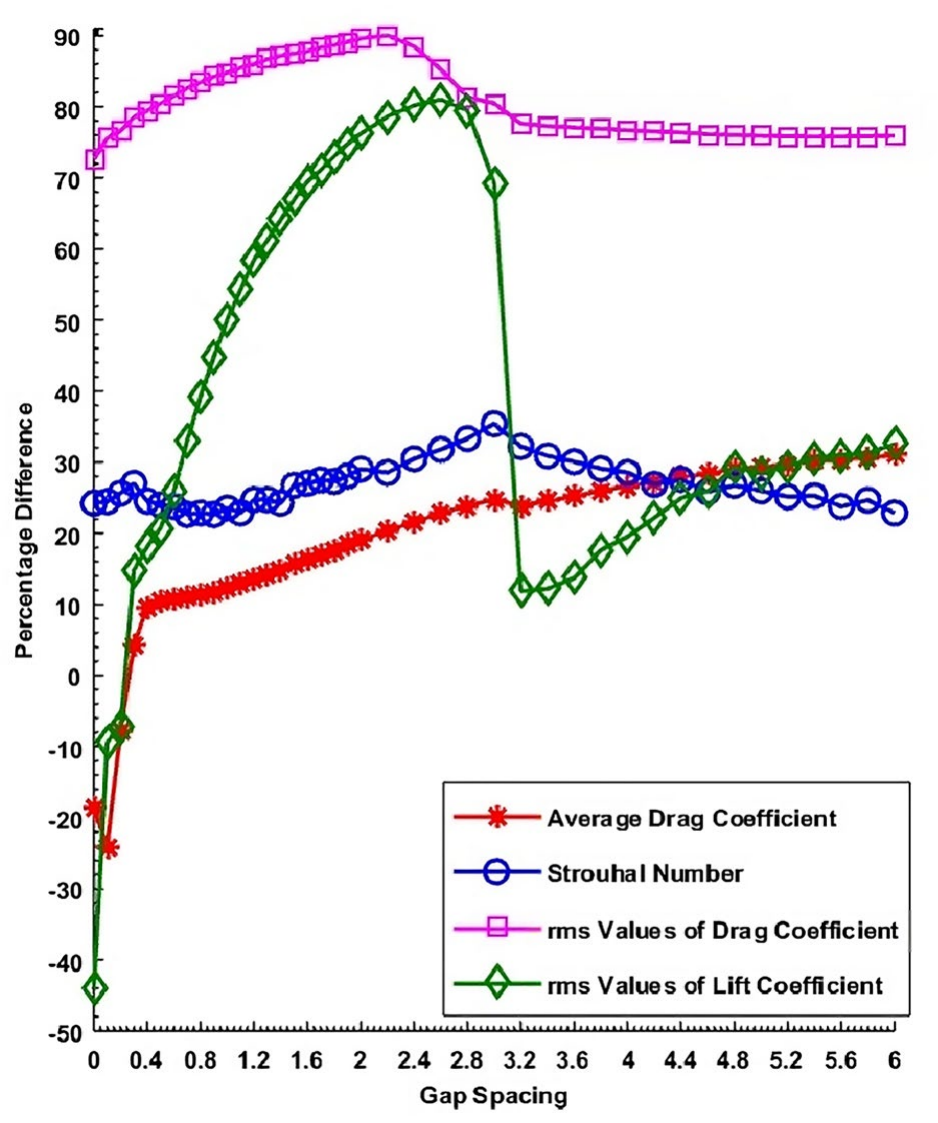
Percentage difference values of physical parameters against gap spacing.

The computed percentage difference values against gap spacings are shown in Fig. 11. This Figure shows the negative values of $C{D}_{m}$ in the range $L$ = 0 to 0.2 indicating that the $C{D}_{m}$ values of cylinders with control cylinders increase as compared to SCWC values. This means that there is an opposite effect of control cylinders on the main cylinder which mainly occurs due to the narrow gap between the main and control cylinders. The positive values show a reduction in $C{D}_{m}$ due to control cylinders. The minimum reduction due to the attachment of control cylinders can be observed at $L$ = 0.3 which is 4.3114 $\text{\%}$. The maximum reduction can be observed at $L$ = 6 which is 31.0244 $\text{\%}$. It is important to mention here that at $L$ = 0.3 the SLR flow regime was observed and at $L$ = 6 the IS flow regime was observed. In the case of the St, it can be observed that the $St$ of the main cylinder with control cylinders reduce at all gap spacing values. The minimum reduction can be observed at $L$ = 0.7 and the maximum reduction can be observed at $L$ = 3 which are 22.6611 $\text{\%}$ and 35.4123 $\text{\%}$, respectively. At these spacing values, SLR and SFDF flow regimes were observed. Since the $St$ values are directly linked with the shedding frequency of vortices. So from the behavior of Strouhal number and average drag force, it can be inferred that at smaller spacing values the drag force increased due to control cylinders while vortex shedding frequency reduced. Also if we compare the percentage of reduction in these parameters we can see that the percentage of reduction in shedding frequency is more as compared to drag force due to control cylinders. The percentage difference graph in $C{D}_{rms}$ against the gap, spacing reveals that the minimum percentage difference of $C{D}_{rms}$ value occurs at $L$ = 0 which is 72.5416 $\text{\%}$ where the flow structure was named SBR. In the case of $C{D}_{rms}$, the highest reduction found in the SFDF flow regime at $L$ = 2.2, which is 90.0517 $\text{\%}$ and it is also the highest among all parameters. The percentage difference in $C{L}_{rms}$ versus gap spacing shows that in the range of gap spacing from 0 to 0.2 the negative values of $C{L}_{rms}$ percentage difference appears indicating the increment in $C{L}_{rms}$ by introducing the control cylinders as compared to SCWC. With the further increment in the gap between the main cylinder and the control cylinder the $C{L}_{rms}$ reduces. The minimum reduction can be found at $L$ = 3.2 which is 11.8724 $\text{\%}$ and the maximum reduction of 81.0040 $\text{\%}$ occurred at $L$ = 2.6 where IS and SFDF flow regimes were observed, respectively.

In general from Fig. 11 it can be concluded that physical parameters like $C{D}_{rms}$, $C{L}_{rms}$ and $St$ is controlled significantly by small control cylinders in the SFDF flow regime range 1 $\le$ $L$ $\le$ 3. While a lesser reduction in IS flow regime indicates the weakening effect of control cylinders. Furthermore, in the case of SBR flow the $C{D}_{m}$ and $C{L}_{rms}$ increase instead of reduction after introducing the control cylinders which can be attributed to strong wake interference impact at smaller gap spacing.

### Applications & practical implications of the findings

Our study focuses on investigating the hydrodynamic behavior of control cylinders in the low Reynolds number regime (Re = 100) using advanced computational fluid dynamics (CFD) simulations. While the manuscript may not have explicitly highlighted the significance and practical implications of our findings, we recognize the importance of elucidating this aspect to ensure the relevance of our research to real-world engineering challenges.

Firstly, our research contributes to addressing fundamental questions related to flow control and fluid dynamics in engineering systems operating at low Reynolds numbers. Understanding the flow behavior in this regime is crucial for various applications, including microfluidics, biofluidics, and low-speed aerodynamics. By providing detailed insights into the hydrodynamic interactions and flow characteristics of control cylinders at low Reynolds numbers, our study lays the groundwork for optimizing the design and performance of devices in these fields.

Furthermore, the findings from our research have direct practical implications for engineering applications requiring precise flow control and manipulation. For instance, in microfluidic systems, where laminar flow conditions prevail, the ability to accurately predict and control fluid flow patterns is essential for achieving efficient mixing, particle separation, and chemical reactions. Our study identifies key flow phenomena, such as vortex shedding behavior and the influence of viscous effects, which are critical for designing effective control strategies in microfluidic devices.

Additionally, our research contributes to advancements in the field of flow control and optimization techniques. By conducting a comprehensive numerical analysis and parametric studies, we provide guidelines for optimizing the design parameters of control cylinders to enhance flow control effectiveness and minimize energy losses. These insights are valuable for improving the performance and efficiency of engineering systems operating in low Reynolds number environments, such as underwater vehicles, small-scale propulsion systems, and biomedical devices.

In summary, while the manuscript may have lacked clarity in explaining the significance and practical application of our research to engineering problems, we believe that our findings have important implications for addressing real-world engineering challenges and advancing the field of fluid dynamics. By elucidating the hydrodynamic behavior of control cylinders in low Reynolds number flows, our study contributes to enhancing our understanding of fluid dynamics in engineering systems and offers practical insights for improving device performance and efficiency.

## Conclusion

In conclusion, this study utilized the lattice Boltzmann method to investigate the influence of control cylinders on the flow past a square cylinder. Through comprehensive numerical simulations and parametric studies, several key findings were obtained.

Firstly, the results revealed that by adjusting the rotational speed of the control cylinders, significant changes in the flow characteristics were observed. Specifically, the drag coefficient, lift coefficient, and root mean square lift coefficient were notably affected, leading to alterations in the downstream flow patterns.

Furthermore, the analysis identified four distinct flow regimes based on the gap spacing between the main and control cylinders. These regimes, namely the solo body regime, shear layer reattachment, suppressed fully developed flow, and intermittent shedding, were characterized by specific gap spacing ranges. This categorization provides a clear framework for understanding the complex flow behavior in such configurations.

One of the novel aspects of this study lies in its detailed investigation of the flow phenomena using a parametric approach. By systematically varying parameters such as the gap spacing and rotational speed of the control cylinders, a comprehensive understanding of their impact on the flow was achieved. Additionally, the inclusion of L1 and L2 distances provided valuable insights into the spatial distribution of flow features around the square cylinder.

Moreover, the study addressed the limitations of previous research by considering a two-dimensional analysis, thereby providing specific insights into two-dimensional flow scenarios. This focused approach allowed for a detailed examination of flow characteristics, highlighting the importance of considering dimensionality in flow simulations.

Overall, this study contributes to the existing body of knowledge by offering novel insights into the flow past a square cylinder with control cylinders. The findings not only enhance our understanding of flow control mechanisms but also have potential implications for various engineering applications, including aerodynamics and fluid dynamics.

The 2D numerical computations were carried out for analyzing the effects of small control cylinders on the flow characteristics of a square cylinder by using LBM. The small control cylinders were positioned upstream and in the wake of the main cylinder. Gap spacing between the main cylinder and control cylinders was varied from 0 to 6 at $Re$ = 100. The results were displayed and discussed in terms of vorticity contours, streamlines visualization, time-dependent drag and lift coefficients, and energy spectrums. Also the effect of control cylinders on physical parameters such as $C{D}_{m}$, $St$, $C{D}_{rms}$ and $C{L}_{rms}$ was discussed briefly. Furthermore, the percentage difference values were obtained by comparing these parameters’ values to those of a single square cylinder without control cylinders.

### The key findings of this work are summarized below:

1. Four different flow regimes were found by varying the gap distance between the main and control cylinders: (i) single body regime (SBR) at 0 $\le$ $L$ $\le$ 0.2, (ii) shear layer reattachment (SLR) at 0.3 $\le$ $L$ $\le$ 0.9, (iii) suppressed fully developed flow (SFDF) at 1 $\le$ $L$ $\le$ 3 and (iv) intermittent shedding (IS) at 3.2 $\le$ $L$ $\le$ 6.
2. The increment in gap spacing between cylinders showed a reduction in the amplitude of fluctuating lift coefficient at all gap spacings. While the rms value of the drag coefficient was found to be the highest reduced value in terms of percentage differences.
3. The analysis of the energy spectrum revealed that vortex shedding is characterized by a single primary frequency. Also, the percentage of reduction in shedding frequency was found to be more as compared to the average drag force due to control cylinders.
4. The physical parameters like $C{D}_{rms}$, $C{L}_{rms}$ and $St$ was significantly controlled by small control cylinders in the SFDF regime range 1 $\le$ $L$ $\le$ 3. While a lesser reduction in IS flow regime indicated the weakening effect of control cylinders.
5. The maximum reduction in $C{D}_{m}$, $St$, $C{D}_{rms}$ and $C{L}_{rms}$ due to control cylinders was observed to be approximately 31 $\text{\%}$, 35 $\text{\%}$, 90 $\text{\%},$ and 81 $\text{\%}$, respectively. Also, results indicated that in terms of the flow regimes generally the maximum reduction was achieved in the case of the SFDF regime.

Future research directions may involve extending the study to three-dimensional simulations to further explore the effects of control cylinders in more complex flow scenarios. Additionally, experimental validation of the numerical results could provide valuable confirmation of the observed flow phenomena.

In summary, this study underscores the significance of flow control strategies in mitigating aerodynamic drag and enhancing the performance of bluff bodies. By elucidating the underlying flow mechanisms, this research contributes to advancements in flow control techniques and lays the groundwork for future studies in the field.

## Data Availability

Data will be available on request by contacting the corresponding author, Dr. Ahmed Refaie Ali, via ahmed.refaie@science.menofia.edu.eg, OR via Dr. Afraz Hussain Majeed at afraz@ujs.edu.cn.
